# Undergraduate Research at Primarily Undergraduate Institutions: Current State and Dilemmas

**DOI:** 10.1021/acsomega.5c08717

**Published:** 2025-11-06

**Authors:** Maira Faisal

**Affiliations:** Department of Biological Sciences and Department of English, 3897 Northern Kentucky University, Highland Heights, Kentucky 41076, United States

## Abstract

The growth of undergraduate research (UGR) in popularity and depth in the past 25 years is greatly owed to primarily undergraduate institutions (PUIs). Studies have shown that laboratories at academic institutions are broadly either student- or research-focused: the former is more common in PUIs, and the latter in research-intensive institutions. However, the student-oriented approach is more beneficial to the student—and, in the long run, the scientific community. The student-centered approach combats the unsustainable shift in scientific academia toward productivity over progress, an intensifying issue in research. The shift encourages researchers to resort to tools like artificial intelligence instead of working thoroughly and incrementally toward new knowledge. With young researchers inheriting this mindset, we worsen our understanding of the knowledge we have and that which we could learn. As an undergraduate student majoring in both STEM and the humanities as well as participating in biochemistry UGR at a PUI, I believe part of these problems arises from a lack of information literacy (IL). The foundational concepts of IL teach the iterative process of research and instill a respect for the creation of information. These ideas reflect the supportive culture of PUIs that initially allowed students (and, in turn, their institutions) to flourish within research.

## Graphical abstract

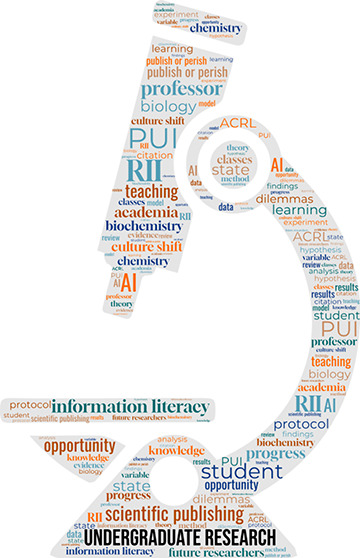

## Introduction

Broadly speaking, there are two institutional classifications that can be made regarding research at universities. Primarily undergraduate institutions (PUIs) have a cultural focus on serving undergraduates.^1^ On the other hand, research-intensive institutions (RIIs) specialize in awarding doctoral degrees and, consequently, have a great emphasis on research; they are the places dubbed R1 and R2 by the Carnegie Classification, an authority on higher education in the United States.^1,2^ It should be noted that RIIs can have more undergraduate students than graduate ones, but the distinction comes from their prioritization of the education of graduates over undergraduates.^1^ Essentially, the defining characteristic is the culture.

PUIs were and are a large part of why undergraduate research (UGR) has risen in prevalence. Twenty-five years ago, research was more so reserved for graduate students,^3^ and this is still often the case at RIIs. But PUIs have been integral in changing this. When evaluating the impact of the faculty–student relationship in the past, the greater focus on undergraduate understanding in research at PUIs demonstrated greater triumphs.^3^ This widened the scope of UGR in the sciences as well as encouraged its spread through nonscience disciplines (such as engineering and the humanities, which did not invoke student involvement in research as naturally).^3^ Thus, as UGR has increased, so have its successes at both the individual and university level, which are worth highlighting and celebrating.^3–5^

My undergraduate institution, Northern Kentucky University (NKU), is a PUI, and I have seen and benefited from the effects of this classification firsthand. No matter the subject, most professors interact with each student in their courses, a connection that extends to the realm of UGR. I say this as a student majoring in multiple disciplines (biological sciences and English) and someone who has been in two different research laboratories (one led by a biology professor and the other by a chemistry professor). Because of circumstances more than any fault of the faculties, this type of connection is rarer in RIIs; the support of postdoctoral researchers and graduate students may remedy this, but only partially. Also, my first experience with UGR was through a program that aimed to introduce first-year students to research. It permitted me to learn about the research of dozens of faculty members, write my first literature review, and join my first research lab. Several of my friends participated in similar programs to kickstart their journeys; some programs were major-specific, while others focused on those who had not yet participated in research. I doubt that research would have felt like a possibility for many of us at NKU if there had not been such a clear institutional emphasis on UGR.

However, in the present day, we are at a wonderful but critical point with UGR, especially in PUIs. The cost of the cultural shift in science (and beyond) toward results over knowledge is beginning to reveal itself.^6,7^ Students cannot reach their full potential in UGR if they do not understand the research process and are not given the tools to do so. It is imperative that these systemic issues are tackled head-on with an earnest determination, not “solved” with resignation through shortcuts like artificial intelligence (AI). Teaching students the principles of information literacy (IL) could be worthwhile, as they can make research more approachable and intelligible.

## UGR, Current State

Studies show that students benefit when faculty have a student-oriented approach to UGR—a research-oriented approach actually hinders them. This discrepancy is easiest to grasp when seeing that these approaches equate to being process- versus product-oriented, respectively.^3^ For an undergraduate, a person unaccustomed to both the process and the content of research, being told of a starting and ending point is not conducive to success. Given a protocol, they may be able to perform an experiment, but they are unlikely to be able to articulate the purpose of the technique or of their day’s work in the context of the larger research project.^3,8^ Typically, PUIs are student-oriented, while RIIs are research-oriented, reinforcing the importance of PUIs in terms of UGR.^3^

Further, the discrepancy in impact is because student-oriented approaches provide students with more opportunities and attention. This ultimately leads to a deeper comprehension of the given subject. It also creates a feedback loop: the investment in undergraduates makes for better students who bolster their universities’ reputations through improved academic and research endeavors.^3,4^ For instance, participating in UGR for one or more semesters corresponds to a statistically significantly higher GPA even when adjusted for students’ SAT scores and sex. In fact, a student’s GPA increases with the greater number of semesters that they participate in UGR.^5^ This aligns with how students under a process-oriented approach are reported to have greater leadership abilities, cognitive enhancements, and higher program satisfaction; these characteristics are negatively associated with a product-oriented approach.^3,5^ Correlation, however, does not equal causation, so inquiry into the matter is needed. Still, it does seem that because they are mentored rather than shepherded by their principal investigator (PI), students in UGR at PUIs are afforded the space to come into their own as young scientists and researchers.^1,3^ With this, they discover their strengths and weaknesses as well as how they can best contribute to the project at hand.

Again, I have experienced this at my PUI. As an example, I took a genetics course with a lab in which we performed a polymerase chain reaction (PCR). A few semesters out from the class, I did not remember how or why I had performed a PCR, only that I had performed one. Conversely, for the research lab I am currently in, I have performed a myriad of PCRs and retained the knowledge surrounding them. This is because, in the class, I was responsible for one sample, while in the lab, I am responsible for a variety of samples and primers for which I adjust the protocol each time. In the class, I loaded one well of the gel, while in the lab, I make, run, and dispose of gels as well as load every sample. Also, through the lab, I have learned that there are different kinds of PCRs, run both analytical and purification gels, and supervised newer lab members during their own PCRs.

This breadth of experience and knowledge with a common scientific technique such as PCR is a result of being part of UGR in a PUI. Joining my lab was a matter of asking my biochemistry professor for his consideration while being in his class. The lab consists solely of undergraduates, many of whom became members through programs like the one I used to join my first lab. The PI attends multiple conferences per semester and ensures each person in the lab has an opportunity to present their work in some way. If anyone has a question, the professor is almost always in a vicinity in which he can physically provide an answer and clarify points. Participating in my lab as much as I have has been possible in large part due to the research environment NKU fosters.

## UGR, Current Dilemmas

While the progress made thus far is admirable, it is important to ensure its continuance. An emerging issue is limiting the influence of academia’s push toward results over knowledge on PUIs. Though budgeting constraints occasionally force PUIs to curb UGR opportunities for the upkeep of teaching loads,^4^ such research limitations at PUIs are well-known by faculty. Per contra, striving institutions, those reaching for greater prestige, ask administrators to dedicate resources and faculty to allocate their time toward results, which are often innovations and journal articles.^2^ These universities can generally be said to be PUIs, as they are striving to raise their Carnegie Classification, a topic that matters to those without the R designation carried by RIIs.^1,2^ In this way, changes in institutional missions, what is called institutional drift, are almost always toward the research-oriented approach.^3^ But acquiescing to the shift lessens the distance between the cultures of PUIs and RIIs, causing PUIs to lose the most unique and useful aspects of their UGR while still not gaining the resources of RIIs.^3,4^ In a similar vein, each college has a context that it is best suited for, based on its location, resources, history, students, and faculty. Advancement is a boon with growing pains, but not every college is meant to be, nor should every one try to be, designated as an R1.

Centering results also does not help the wider scientific community. This push for productivity is not sustainable^6^ and could encourage turning to tools such as artificial intelligence (AI).^7^ In our context, it is more efficient to address how AI, throughout its applications, generally impacts the process of research, from summarizing papers to creating artificial data sets.^7^ Defining AI and its subsets may seem more of a tangent than a true help here. Still, AI can broadly be seen as algorithms that attempt to perform tasks, which humans are currently better at, to mimic human intelligence.^9,10^ Under the umbrella of AI is machine learning, under which is deep learning, under which is deep generative models. The large language models most colloquially addressed as AI are classified as one of many deep generative models.^9,10^

But truly, all of the aforementioned concepts are part of AI, which can generate artificial hallucinations^11^ and often gives researchers a false image of their understanding of a topic.^7^ In a perverse cycle, AI could permit an increase in the volume, though not necessarily quality, of research,^7^ inflating productivity standards that could then have to be met with further AI utilization. But AI can only give an output based on its input, which is preexisting knowledge: any and all gaps, biases, and errors are replicated in what it predicts and generates.^7,11–14^ While it can give simple, if reductive, explanations of complex subjects, it can neither accurately address the minutiae that the majority of research projects involve nor provide the references that could serve as jumping-off points.^7,11,14^ Cognitively diverse teams lead to better problem solving, while demographically diverse ones lead to, citations-wise, more impactful research;^7,15^ yet these diversities are not properly expressed by AI.^7,12^ And when exposed to information about a subject, people commonly mistake others’ cognizance of the concept as their own, an issue exacerbated with AI.^7^ Having limitations as humans reinforces slower, steadier progress: this is not a flaw of research but a safeguarding characteristic. The human checks and balances of the research process, from proper sampling to peer review, exist to prevent the scientific monoculture that AI threatens to bring to fruition.^7,13^ Movement itself is not progress, and to date, our natural limitations have finetuned our excess movement until it was actual, honest, and useful advancement. This is because science is about innovation, not about the number of papers one publishes.^6,13^

I believe this “publish or perish” mentality^6^ is due, in part, to a lack of IL in scientific communities.^16^ As someone majoring in both the humanities and STEM, in my freshman year, I did an IL fellowship at my PUI’s academic library. During this time, I learned of the *Frameworks for Information Literacy for Higher Education* developed by the Association of College and Research Libraries (ACRL).^17^ The frameworks are listed below, along with definitions coalesced by me for student use during my fellowship.

1. Authority Is Constructed and Contextual
  - The authoritativeness of an author depends on the context of what they wrote and what one is trying to write; it can be constructed in the sense that different communities recognize authority differently.
2. Information Creation as a Process
  - Researching, to create or arrive at new information, is a nonlinear process that requires putting together many puzzle pieces.
3. Information Has Value
  - Information is inherently valuable, whether it be used for educational, commercial, or other purposes.
4. Research as Inquiry
  - Research can be simplified into the notion of asking questions (and later expanded to finding the answers to said questions in a valid, thoughtful manner).
5. Scholarship as Conversation
  - Research, as a field, serves as a sort of conversation between scholars and their findings, musings, and further questions.
6. Searching as Strategic Exploration
  - Searching for the answers to research questions is a complicated process that often results in an exploration of the topic at hand as well as the discovery of the sought-after answer.

To be a good researcher, one needs to have a good foundation of knowledge in their subject area but also of the conduct of research itself.^15,17–19^ The ACRL therefore generated these concepts to act as a structure for IL across academic disciplines. Students may implicitly understand some frameworks’ ideas, but knowing them explicitly elucidates the research process so that their efforts in UGR go further.^15,18,19^ Scientific IL specifically is neglected because scientists frequently believe their needs will not be met, yet this is what makes librarians’ comprehension of scientific IL needs worse. This circles back to affirm the scientists’ preconceived notions of IL.^15,16,18^

The frameworks note the iterative process of research and touch on respecting research as it relates to expanding knowledge at the individual and community level. This description of research as nonlinear highlights the nature of its process before a student, acquainted with the directness of course laboratories, has the opportunity to be confused or frustrated by it in UGR.^17,19^ Meanwhile, this respect does not allow someone to accept the culture of “publish or perish” or the excessive use of AI; this heightens the caliber of a person’s work because when writing and organization—the very means of communication and critical thinking—are portrayed as unimportant tasks to be passed off, we limit ourselves.^12,15,17,19^ More concretely, mastery of IL is also correlated with higher grade point averages and general student success.^16,18^ Just as my praise for UGR at PUIs was, my caution expressed here is reinforced by my personal experience. I have witnessed a myriad of students, mostly in STEM, paint results, frequently obtained through greater labor and AI use, as a panacea when they are not. As pertinent as results are, they do not matter if one cannot express them with the nuance they, their practices, and their potential directions are due.

Ultimately, UGR is the basis upon which the future of research rests, as the undergraduates of today will grow into the experts of tomorrow.^1,3,4^ It is the job of all institutions, but particularly PUIs, to certify that the next generation inherits the skills and uprightness required for their positions and duties as researchers.

## References

[1] Dahlberg C. L., King-Smith C., Riggs B. (2021). Building a Laboratory at a Primarily
Undergraduate Institution (PUI). BMC Proceedings.

[2] Ellis, M. Core Tensions: An Exploratory Case Study of Faculty Work at a Striving Institution. Ph.D. Dissertation, Southern Methodist University, 2019. https://scholar.smu.edu/simmons_depl_etds/3.

[3] Malachowski M. R. (2020). Reflections
on the Evolution of Undergraduate Research at Primarily Undergraduate
Institutions Over the Past 25 Years. CUR Quarterly.

[4] Ball N. D., Gomez M. A., Rempel B. P., Farkas E. R., Makal T. E., Shields G. C., Parish C. A., Tresca B. W., McGinitie E. G. (2023). Conducting
Research at Primarily Undergraduate Institutions. Cell Reports Physical Science.

[5] Fechheimer M., Webber K., Kleiber P. B. (2011). How Well
Do Undergraduate Research
Programs Promote Engagement and Success of Students?. CBE Life Sci. Educ.

[6] Hanson M. A., Barreiro P. G., Crosetto P., Brockington D. (2024). The Strain
on Scientific Publishing. Quantitative Science
Studies.

[7] Messeri L., Crockett M. J. (2024). Artificial Intelligence and Illusions of Understanding
in Scientific Research. Nature.

[8] Dewey J., Evers A., Schuchardt A. (2022). Students’ Experiences and
Perceptions of the Scientific Research Culture after Participating
in Different Course-Based Undergraduate Research Experience Models. CBE Life Sci. Educ.

[9] Strobel, G. ; Banh, L. ; Möller, F. ; Schoormann, T. Exploring Generative Artificial Intelligence: A Taxonomy and Types. Proceedings of the 57th Hawaii International Conference on System Sciences; 2024; p 4546. 10.24251/HICSS.2024.546.

[10] Ertel, W. Introduction. Introduction to Artificial Intelligence; Springer Nature, 2024; pp 1–23. 10.1007/978-3-658-43102-0.

[11] Alkaissi H., McFarlane S. I. (2023). Artificial Hallucinations in ChatGPT:
Implications
in Scientific Writing. Cureus.

[12] Noy S., Zhang W. (2023). Experimental Evidence on the Productivity Effects of Generative Artificial
Intelligence. Science.

[13] Thorp H. H. (2023). ChatGPT
Is Fun, but Not an Author. Science.

[14] Van
Dis E. A. M., Bollen J., Zuidema W., Van Rooij R., Bockting C. L. (2023). ChatGPT: Five Priorities for Research. Nature.

[15] Valladares L. (2021). Scientific
Literacy and Social Transformation: Critical Perspectives About Science
Participation and Emancipation. Sci. & Educ.

[16] Witherspoon R., Taber P., Goudreau A. (2022). Science Students’ Information
Literacy Needs: A Survey of Science Faculty on What and When Each
Skill Is Needed. College & Research Libraries.

[17] Association of College and Research Libraries . Framework for Information Literacy for Higher Education; 2015. https://www.ala.org/sites/default/files/acrl/content/issues/infolit/Framework_ILHE.pdf.

[18] Perry H. B. (2017). Information
Literacy in the Sciences: Faculty Perception of Undergraduate Student
Skill. College & Research Libraries.

[19] Grogan K. E. (2021). Writing
Science: What Makes Scientific Writing Hard and How to Make It Easier. Bulletin of the Ecological Society of America.

